# Correction: An integrated group decision-making method under q-rung orthopair fuzzy 2-tuple linguistic context with partial weight information

**DOI:** 10.1371/journal.pone.0316843

**Published:** 2024-12-30

**Authors:** Fatima Abbas, Jawad Ali, Wali Khan Mashwani, Muhammad I. Syam

In Author Contributions, Muhammad I. Syam should have not been written as the one who acquired financial support for the project leading to publication (**Funding acquisition**) but as the one who developed the methodology (**Methodology**).

The authors, Fatima Abbas, Jawad Ali, Wali Khan Mashwani, Muhammad I. Syam should be noted as contributing equally to this work.
